# Utilization of Leather Waste Fibers in Polymer Matrix Composites Based on Acrylonitrile-Butadiene Rubber

**DOI:** 10.3390/polym13010117

**Published:** 2020-12-30

**Authors:** Le Thuy Hang, Do Quoc Viet, Nguyen Pham Duy Linh, Vu Anh Doan, Hai-Linh Thi Dang, Van-Duong Dao, Pham Anh Tuan

**Affiliations:** 1 School of Textile—Leather and Fashion, Hanoi University of Science and Technology, Hanoi 100000, Vietnam; hanglt1983@gmail.com; 2 Hung Yen University of Technology and Education, Hung Yen 160000, Vietnam; 3 Center for Polymer Composite and Paper, Hanoi University of Science of Technology, Hanoi 100000, Vietnam; vietzat@gmail.com; 4 Faculty of Materials Science and Engineering, Phenikaa University, Hanoi 100000, Vietnam; 5 Faculty of Environmental Sciences, VNU University of Science, Vietnam National University, 334 Nguyen Trai, Hanoi 100000, Vietnam; hlinh.93@gmail.com; 6 Faculty of Biotechnology, Chemistry and Environmental Engineering, Phenikaa University, Hanoi 100000, Vietnam; fattuan@vicostone.com

**Keywords:** waste leather, acrylonitrile butadiene rubber, tensile strength

## Abstract

In this study, we present the fabrication of nitrile butadiene rubber/waste leather fiber (NBR/WLF) composites with different weight percentages of WLF and NBR (0/100, 20/80, 30/70, 40/60, 50/50, 60/40 *wt*/*wt*). WLF was prepared by cutting the scrap leathers from the waste product of the Vietnamese leather industry. Subsequently, in order to make the short fibers, it was mixed by a hammer mill. The characteristics of WLF/NBR composites such as mechanical properties (tensile strength, tear strength, hardness), dynamic mechanical properties, toluene absorption, and morphology were carefully evaluated. As a result, the tensile strength and tear strength become larger with increasing WLF content from 0 to 50 wt% and they decrease when further increasing WLF content. The highest tensile strength of 12.5 MPa and tear strength of 72.47 N/mm were achieved with the WLF/NBR ratio of 50/50 wt%. Both hardness and resistance of the developed materials with toluene increased with increasing WLF content. The SEM results showed a good adhesion of NBR matrix and the WLF. The increasing of storage modulus (E’) in comparison with raw NBR showed good compatibility between WLF and NBR matrix. This research showed that the recycled material from waste leather and NBR was successfully prepared and has great potential for manufacturing products such as floor covering courts and playgrounds, etc.

## 1. Introduction

The generated waste products from the leather industry are annually causing a serious problem for the environment and human health [1,2]. One of the main issues is the presence of noxious chemicals, especially heavy metal ions, which are quite complicated to deal with, such as Cr (III) and Cr (VI) after the tanning process [2,3]. Therefore, recycling such materials is one of the largest concerns of society. Leathers made of animal skin are found as a natural polymer. It is well known that the leather structure contains many layers such as grain, corium, and flesh layer, of which the majority of thickness and strength is the corium layer, which consists of a long fibrous wave of collagen fibrils in the fiber network layer [4]. In general, the complex layered structure of common collagen and collagen of leather after the tanning process was studied and reported in many works [5,6,7,8]. Due to the presence of amine groups [6] and carboxyl groups [8] in the collagen structure, it can be expected that the leather can be highly compatible with the polar polymer matrix. Furthermore, the natural crosslinking formed by the interaction between these groups on the fibril chain through hydrogen bond plays an important role in the mechanical strength of fibers.

Nitrile butadiene rubber (NBR) is one of the most common polar synthetic rubbers and is widely used in industry due to the high resistance to oil, organic solvent, and chemicals. Most of the properties of NBR directly depend on the content of the nitrile group. Higher nitrile content imparts higher tensile properties, wear resistance, stiffness, and especially resistance to oil [9]. Nevertheless, NBR without filler has poor mechanical properties. Therefore, the seeking of replaceable fillers such as carbon black or other inorganic additives for traditional fillers is extremely important.

In recent years, the trend of developing bio-composites from recyclable waste resources and natural fiber has attracted many researchers [10,11,12,13,14,15,16,17,18,19,20]. Due to the cost-effective and renewable property, the use of leather short fibers, especially fibers from the industrial waste resource, is also feasible. Many previous works showed that leather could be incorporated with NBR as a filler factor. Salwa H. El-Sabbagh et al. investigated the influence of untreated and treated leather dust waste on the incorporation process with NBR [20]. The results illustrated that the chemical treatment of leather by ammonia and sodium formate has impacts on tensile strength, Young’s modulus, and rheological properties. K.Chronska-Olszewska et al. also studied NBR and Carboxylated Nitrile Rubber reinforced by leather shaving-dust [15]. Accordingly, the formation of composition resulted in slightly changing viscosity as well as biodegradation. In the research on the incorporation of leather fibers with different rubbers, I. Shabani et al. concluded that the compatibility of leather fibers with polar rubber is higher than that with nonpolar rubber [19]. Although many studies have been reported about the incorporation of waste leather fiber (WLF) with different kinds of rubber matrix [10,12,17,19], and plastic [21,22,23] as well, the research of using a higher amount of waste leather in the composite has been rarely published.

In this paper, we presented the reuse of WLF in the fabrication of WLF/NBR composites with different weight percentages of WLF and NBR (0/100, 20/80, 30/70, 40/60, 50/50, 60/40 *wt%*). The scrap leathers were ground to fibers before introducing to NBR with a higher proportion to maximize the amount of waste material. Furthermore, we also carefully investigated all properties of the composites such as mechanical properties (tensile strength, tear strength, hardness), dynamic mechanical analysis (DMA) properties, solution absorption, and morphology. This strategy is simple and efficient for fabricating WLF/NBR composites from reusing WLF and its application in the floor covering courts and playgrounds, etc.

## 2. Materials and Methods

### 2.1. Materials

Acrylonitrile butadiene rubber was bought from Kumho, Seoul, Korea with 33% AN content. The corium layer of waste leather was collected after the manufacturing process from the Vietnamese leather factory (Hung Yen, Viet nam). Other chemicals for the vulcanization process, such as stearic acid, zinc oxide, sulfur, and accelerator were supplied from Henan Kingway chemical co. ltd, China.

### 2.2. Drying and Size-Reduction of the Corium Layer

The leather was dried at 80 °C under the air circulator oven for 2 h to remove moisture. After that, the corium leather was size-reduced to a length of 4.5–10 mm width of 0.1–0.2 mm by using a hammer mill at 2000 rpm of rotor speed, with six rows of hammers and 40 mesh to form WLF.

### 2.3. Preparation of Leather/NBR Composite

The detailed formulation of WLF/NBR is given in Table 1. The WLF/NBR composites with different leather contents and additives were carried out using Labo Plastomill 4M150 internal mixer (Japan). The mixture was vulcanized under conditions of 150 °C and 10 MPa.

### 2.4. Characterization

#### 2.4.1. Characterization of Vulcanized Material

The properties of the vulcanization process were determined at 150 °C by using Rotorless Rheometer RLR-4-Toyoseiki (Japan). Other parameters such as maximum (MH) and minimum (ML) torque, scorch time (*ts*_2_), and optimum cure time (*tc*_90_) were obtained from the graph. The cure rate index (CRI) was calculated by the equation: [24]

$$\mathrm{CRI}\;(\min^{-1})\;=\;\frac{100}{\left(tc_{90}-ts_{2}\right)}$$

#### 2.4.2. Mechanical Properties

Tensile and tear properties of the vulcanized WLF/NBR were investigated by using INSTRON 5582 testing machine (USA) with 500 mm/min of crosshead speed. The dumbbell-shaped specimens were prepared by a gripping tool with the 2-mm-thick films following ASTMD-D412-D. Likewise, the specimens for tear strength measurement were also prepared according to ASTMD-624-C. The samples were placed into stability conditions at room temperature for about 24 h before testing.

#### 2.4.3. Morphology of the Developed Composites

Scanning Electron Microscopy (SEM, JEOL JSM 6360 LV equipment, Japan) was used to define the morphology of composite with the fractured surface after the tensile test. The samples were coated with platinum and the accelerating voltage was set at 20 kV.

#### 2.4.4. Solution Absorption

The toluene absorption was carried out as ISO 1817-2005 standard. The toluene absorption content of composite material was calculated as expression:

$$\Delta m=\frac{m_{1}-m_{0}}{m_{0}}\times 100\%$$

where:

Δm is toluene absorption content of composite material (%)
*m*_1_ is the weight of toluene saturated WLF/NBR composite (g)
*m*_0_ is the weight of the initial WLF/NBR composite (g)

#### 2.4.5. Dynamic Mechanical Analysis

Dynamic mechanical properties of WLF/NBR were measured by using a dynamic mechanical analyzer (Mettler Toledo, DMA 8000, USA), which was supplied by Mettler-Toledo (M) Sdn. Bhd. The samples were subjected to a cyclic tensile strain with a force amplitude of 0.1 N at a frequency of 1 Hz. Storage modulus (E′), loss modulus (E″), and mechanical loss factor (tan d) were determined in the temperature range from −80 °C to 50 °C at a heating rate of 2 °C/min.

## 3. Results and Discussion

### 3.1. Effect of WLF on Composite Vulcanization Behavior

**Figure 1:**
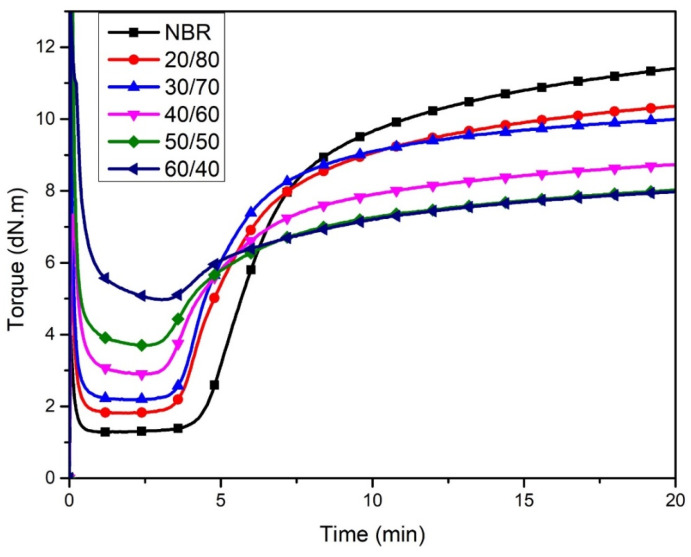
Rheograph of various WLF/NBR ratio.

Vulcanization behavior of WLF/NBR composite with various WLF content is shown in Figure 1 and the obtained results are summarized in Table 2. It can be seen that the minimum torque ML, which indicates the initial viscosity and process-ability of composite, increased continuously from 0.129 N.m for NBR to 0.498 N.m for 60/40 WLF/NBR composite corresponding to the increase of WLF content. The reason could be that WLF in composite material interacted well with the NBR matrix and the rigid fibrous structure of WLF obstructed the rotation of the mixing shaft, leading to the increasing of processing torque. The obtained results are in good agreement with Ferreia et al.’s reported [10].

In contrast, there was a downward trend in the maximum torque MH when the content of WLF increased. Generally, the maximum torque MH reflects the cross-link density [25]. In this case, it could be the decline in cross-link density between the rubber chains in NBR phase, which may be caused by two main factors. Firstly, the WLF content is lower than that of NBR. Then, when the WLF content increased, the proportion of NBR declines respectively, leading to a decreased amount of polymer molecules reacting in the vulcanization. Secondly, the rigid network of leather fibers also provides the discontinuous matrix that is a limitation of the moving space of polymer molecules during the reaction process. Furthermore, it was found that the scorch time (*ts*_2_) values of the compounds with different WLF/NBR ratios were lower than that of the raw NBR. It could be attributed to the presence of reactive functional groups in leather fibers, which act as activators and accelerate the rate of reaction [26]. This phenomenon was further proved by the calculated parameter of the CRI shown in Table 2. The CRI of composites gradually increased by the upward trend of WLF content. The highest point of 33.11 min^−1^ was reached at 60/40 *wt%* of WLF/NBR ratio. It illustrated that the functional groups available in WLF could be an accelerator affecting not only *ts*_2_ but also optimum time (*tc*_90_) in the vulcanized NBR compound.

### 3.2. Effect of WLF Content on Mechanical Properties of Composite Material

#### 3.2.1. Tensile Properties

**Figure 2:**
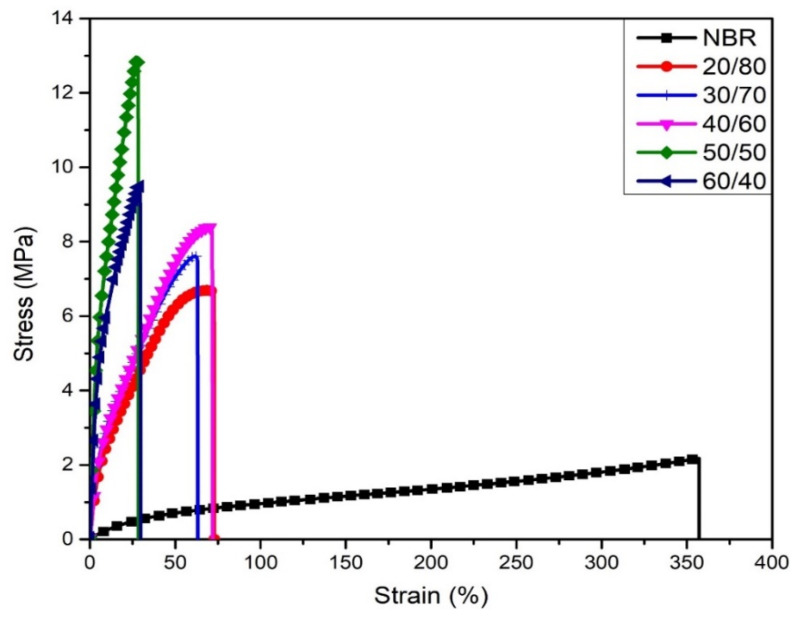
Stress–strain curve for various WLF/NBR ratio.

The effect of WLF content on tensile properties of WFL/NBR composite was described by the stress–strain curve as shown in Figure 2. It should be noted that an increase in stress–strain curve slope means an augment in stiffness of NBR matrix and a decrease in the elongation at break. This may be due to the rigid fibrous structure of the large WLF content. As can be seen in Table 2, the tensile strength tends to increase by the upward trend of leather proportion. The initial tensile strength and elongation at break of raw NBR rubber were 2.18 MPa and 357%, respectively. It was found that the tensile strength increased with increasing WLF content from 20 to 50 *wt%* in the WLF/NBR composite and it decreased with further increasing WLF content in WLW/NBR composite. The stress at break of WLF/NBR reached a maximum value of 12.9 MPa at the ratio of 50/50 *wt%*, which was approximately 6 times higher than that of raw NBR. The improvement of tensile strength firstly may be attributed to the good dispersion of leather in NBR matrix, which allows to significantly enhancing the load transfer of WLF/NBR composite [14]. Secondly, the compatibility of the functional groups in collagen fibrils and nitrile groups in rubber could be a reason for the strong adhesion between the interfacial surface of WLF and NBR matrix when the fibers are introduced to the nitrile rubber [16,19]. Accordingly, with the existence of WLF in the composite, these bonds that mainly are fibers–fibers and fibers–rubber linking also augment, respectively, because of the rise of total linking. However, the further increasing WLF content (>50 *wt%*) leads to the aggregation of leather bundles formed by fiber–fiber bonding, which is the weakness of the composite under the application of external force [21]. With the presence of leather, unlike the trending of tension, the elongation at break decreased dramatically from 357.2% of raw NBR to 28.4% of the composite with the highest content of WLF. It is also related to the large content of fibers on NBR matrix, which contributed to reduce the flexibility of rubber chains and increase the stiffness of the material [27].

#### 3.2.2. Tear Strength

**Figure 3:**
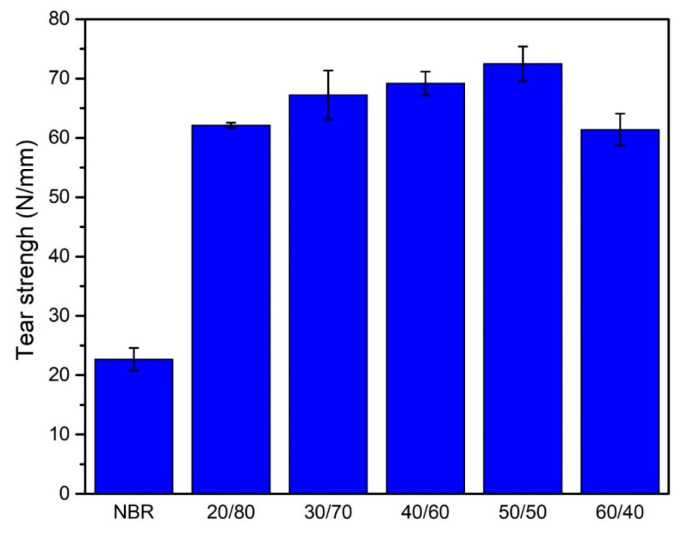
The dependence of WLF loading to tear strength.

Figure 3 exhibits the effect of leather on the tear strength of the NBR composite. The trend of tear strength is quite similar to that of tensile strength when the leather content was raised. For the sample of NBR without leather, the tear strength was 22.7 N/mm. However, there was a three times increase in this value when the WLF/NBR ratio was just 20/80 *wt%*. From 30/70 *wt%* to 50/50 *wt%* of WLF/NBR ratio, the tear strength continuously went up and reached the highest value of 72.47 N/mm at 50/50 *wt%* of WLF/NBR ratio before falling to approximately 60 N/mm when WLF was further added. In comparison with the continuous NBR matrix, the presence of leather fiber considerably enhanced tear resistance. As in many previous publications, tear strength was directly related to crack growth [28,29]. Ferreia et al. have reported that the tear strength was remarkably improved when leather fibers were introduced in both NBR and Styrene-Butadiene Rubber [3]. Mese et al. also archived similar results with the Ethylene Propylene Dien Monomer rubber [17]. As a result, the fibers–fibers linking and fibers–rubber linking were contributed dramatically to the improvement of tear strength.

#### 3.2.3. Hardness

**Figure 4:**
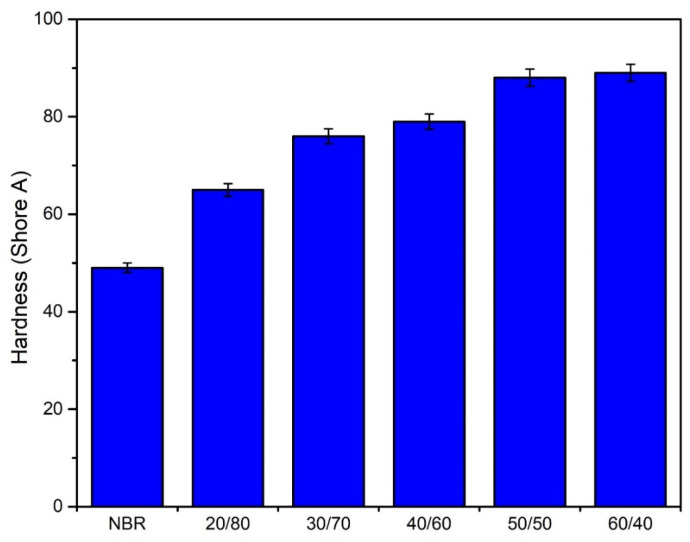
Effect of leather added to NBR on the hardness of composite.

As shown in Figure 4, there was a similar upward trend in the hardness of the material compared to that in tensile properties and tear strength. The rise of hardness with the increasing WLF content could be explained by the mobility reduction of the rubber chain when leather fiber was introduced. This is because collagen fibers have a three-dimensional structure [30]. The bulky dimensions of fibers and the interaction of fiber and rubber lead to the limitation of segmental mobility of NBR chains. Furthermore, the good adhesion between NBR and WLF also restricted the flexible structure of the original material. Elastomer chains hardly moved around each other, also leading to the decrease of elongation at break mentioned in the above section. Consequently, the presence of WLF enhanced the composite’s stiffness. This result is truly accommodated with other previous researches [10,27,31,32].

#### 3.2.4. Effect of WLF Content on Stress Relaxation of Composite Material

**Figure 5:**
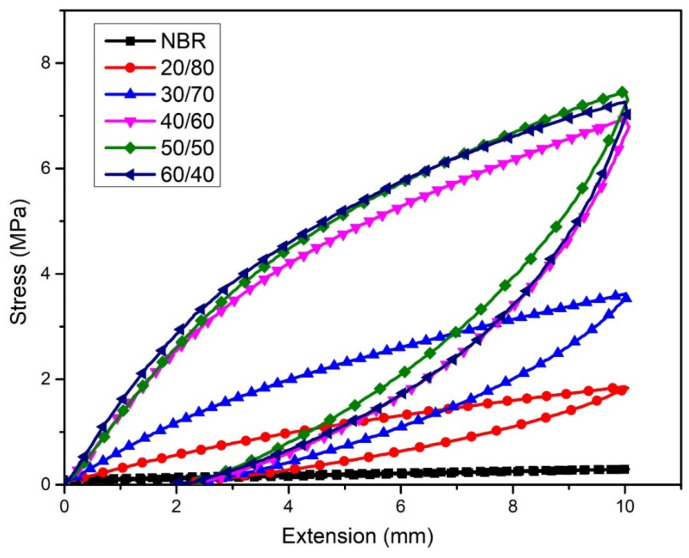
Stress relaxation curve of various WLF/NBR ratios.

The stress relaxation of WLF/NBR composite was estimated by the tensile test principle as shown in Figure 5 and Table 2. In theory, there are three main links in a composite material including matrix–matrix, matrix–reinforcement, and reinforcement–reinforcement. With one normal cycle of loading–unloading stress, the stress relaxation curve of NBR is narrow, as shown in Figure 5. With the addition of WLF, the stress relaxation curve area of WLF/NBR composite increased clearly with the augment of WLF content (Figure 5 and Table 2). The area of each stress relaxation curve is attributed to the energy dissipation in one loading–unloading cycle of composite fabricated from NBR and WLF. The increase of stress by the upward trend of WLF volume at 10 mm elongation also shows the great dispersion and good compatibility of WLF on NBR. Therefore, the differences in the stress relaxation curve area can be considered as the degradation of fibers–fibers bonding. When the external force was applied, this bonding was first stretched and degraded. The unrecoverable energy was dissipated as heat, following the Mullins effect [33].

**Figure 6:**
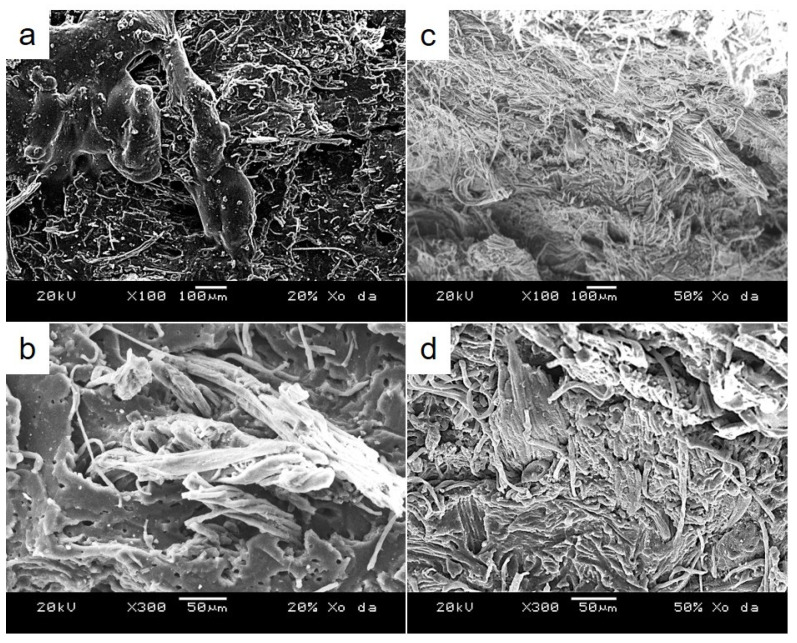
SEM observation of WLF/NBR composite: (**a**,**b**) 20/80 *wt%* and (**c**,**d**) 50/50 *w*/*wt*.

Moreover, the good dispersion of WLF in WLF/NBR composite material was observed through SEM measurements. The SEM images at various magnifications of the tensile fracture surface of WLF/NBR composite (20/80, 50/50 *wt%* of WLF/NBR, respectively) are shown in Figure 6. It can be very clearly seen that leather fibers were dispersed in NBR without orientation. Figure 6a,b exhibit a lot of holes in the rubber surface beside the fiber bundles. This could be a result of pulling out fiber during the tensile test. It is easily found that the position of fiber breakage is near pullout holes, which suggests a weak adhesion between fiber and rubber. However, an increase in WLF content in composites improved the adhesion between WLF and NBR. Indeed, as can be seen in Figure 6c,d, the WLF dispersion was quite good at 50/50 *wt%* of WLF/NBR. The dimension of WLF seems to decrease, which means the WLF bundle was divided into a single fiber. Thus, every single fiber can easily move in NBR matrix, leading to good dispersion of WLF. However, the decline in fiber–fiber interaction also increased the area of the stress relaxation curve when the WLF content increased. Moreover, the strong adhesion between WLF and NBR is evidence of the remarkable increase in the mechanical properties of composites.

### 3.3. Effect of WLF Content on Solution Absorption of WLF/NBR Composite

**Figure 7:**
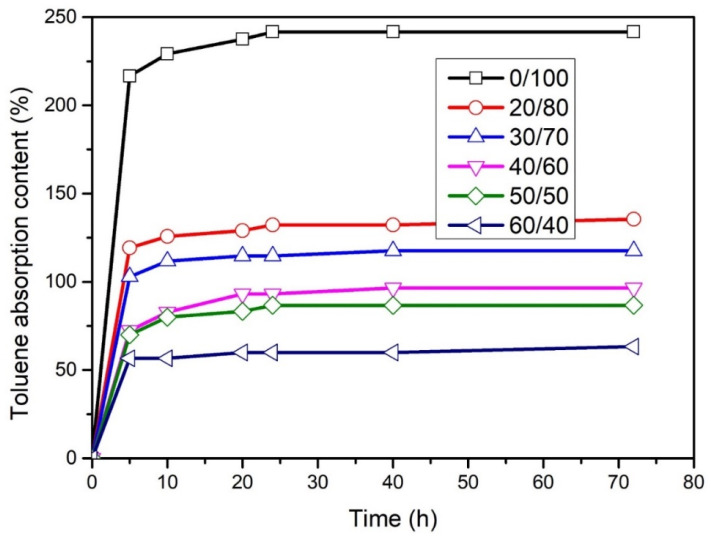
Toluene absorption curve of WLF/NBR composite.

The effect of the WLF/NBR ratio on solution absorption of composites was evaluated with the capacity of absorbing toluene solution. The obtained results are presented in Figure 7. We found that while WLF proportion was increasing, there was a significant decline in toluene absorption content. Several factors, such as high resistance of WLF in a polar solvent, high resistance of nitrile rubber in oil, and aromatic hydrocarbon, can be used to explain this phenomenon. Therefore, with the presence of WLF in the developed composite materials, the solution resistance capacity of NBR was enhanced remarkably. The significant increase of the toluene resistance capacity of WLF/NBR composite when WLF/NBR content augmented also illustrates the good compatibility and dispersion of WLF in NBR matrix. The obtained results indicate the opportunity to develop new material with high oil and polar solvent resistance.

### 3.4. Effect of WLF on Dynamic Mechanical Properties of WLF/NBR Composite

**Figure 8:**
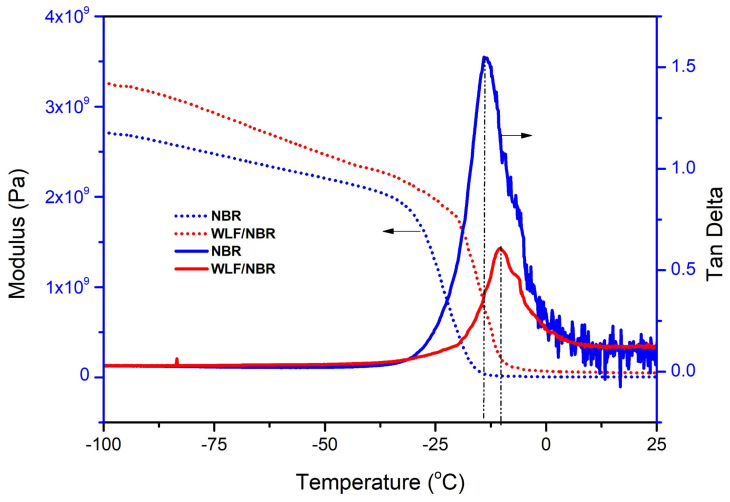
Dynamic mechanical properties of WLF/NBR composite.

The effect of WLF on the dynamic mechanical properties of the material is presented in Figure 8 and Table 3. It was found that the storage modulus of the composite material was much higher than that of raw NBR. It means that the composite material was stiffer than raw NBR and the excellent compatibility between NBR and WLF improves tensile strength, hardness, and tear strength of composites as well. On the other hand, the augment of obtained loss modulus once again explains the increase in the area of stress relaxation curve when the content of WLF in WLF/NBR composite material increased.

## 4. Conclusions

In summary, the utilization of WLF in polymer composite material based on NBR was investigated. The evaluation of the effect of WLF content on mechanical properties showed good compatibility between WLF and NBR, which leads to a significant increase in tensile, tear strength, and hardness of the material. The good compatibility was also proved by the results of SEM, solution absorption, and DMA. The optimum WLF content in the composite material is 50/50 *wt%* of the WLF/NBR ratio, which can provide a stable supply of the WLF for industrial production scale. Note that the developed materials are proposed for high-tonnage applications, as surfaces of the sports facility. The development of WLF/NBR composite is not only a temporary toluene for environmental protection but also opens a new research orientation about the application of recycled waste leather material in the industry.

## Figures and Tables

**Table 1:** The technical parameters for waste leather fiber (WLF)/ nitrile butadiene rubber (NBR) composition.

Ingredients	Part by Weigh of the Rubber (phr)
NBR	100
Stearic acid	1
Zinc oxide	3
TBBS	0.7
Sulfur	2.25
WLF/NBR ratio	0/100; 20/80; 30/70; 40/60; 50/50; 60/40

TBBS: n-tert-butyl-2-benzothiazole sulfonamide.

**Table 2:** Effect of WLF/NBR ratio on vulcanization and tensile properties.

	Composition of WLF/NBR					
	NBR	20/80	30/70	40/60	50/50	60/40
M_L_ (dN.m)	0.129	0.181	0.219	0.290	0.370	0.498
M_H_ (dN.m)	1.049	0.935	0.839	0.785	0.683	0.611
*ts*_2_ (min)	4.76	3.9	3.82	3.46	3.43	3.40
*tc*_90_ (min)	9.82	8.88	8.72	7.47	6.59	6.42
CRI (min^−1^)	19.76	20.08	20.40	24.94	31.64	33.11
Tensile strength (MPa)	2.18	6.7	7.6	8.38	12.9	9.5
Elongation at break (%)	357.2	72.9	63.3	71.8	28.6	28.4
Area under hysteresis (area unit)	---	5.05	11.27	26.35	26.58	29.62

**Table 3:** Storage modulus (E′) and maximum loss modulus (E″_max_) of NBR and WLF/NBR composite with the ratio of 50/50 *wt%*.

Sample	Storage Modulus (Pa)	Maximum Loss Modulus (Pa)
NBR	2.72 × 10^9^	8.29 × 10^7^
WLF/NBR	3.28 × 10^9^	9.34 × 10^7^
